# Associations of C reactive protein to albumin ratio, neutrophil to lymphocyte ratio, platelet to lymphocyte ratio with disease activity in patients with juvenile idiopathic arthritis

**DOI:** 10.1186/s41927-024-00390-x

**Published:** 2024-06-17

**Authors:** Giulia Di Donato, Marina Attanasi, Debora Mariarita d’ Angelo, Saverio La Bella, Armando Di Ludovico, Francesco Chiarelli, Luciana Breda

**Affiliations:** 1 Pediatric Rheumatology Unit, S.S. Annunziata Hospital, Via dei Vestini, 5, Chieti, Italy; 2 Department of Paediatrics, University “G. d’Annunzio”, Chieti, Italy

**Keywords:** Disease activity, C reactive protein to albumin ratio, Juvenile idiopathic arthritis

## Abstract

**Introduction:**

Recent works in the scientific literature reported the role of C reactive protein to albumin ratio (CAR), neutrophil to lymphocyte ratio (NLR) and platelet to lymphocyte ratio (PLR) as biomarkers of disease activity in rheumatic diseases.

**Objectives:**

To investigate the role of CAR, PLR and NLR as potential markers of disease activity in children with non-systemic JIA (nsJIA) and their correlation with the risk of persistent disease activity of flare during follow up.

**Methods:**

Our prospective, cross-sectional study involved 130 nsJIA patients (74 with active disease and 56 with inactive disease according to Wallace criteria) and 62 healthy controls. Demographic, clinical and laboratory data were collected at baseline (T0) and at 3 (T1), 6 (T2), 12 (T3) and 18 months (T4) during follow up. Disease activity was evaluated through Juvenile Arthritis Disease Activity Score (JADAS-27).

**Results:**

At baseline, CRP and CAR were higher in patients than in controls (*p* = 0.046), while no differences were found for NLR and PLR. However, there was no positive correlation between CAR, NLR, PLR and JADAS-27 in JIA patients. To better investigate the role of CAR, NLR and PLR as markers of disease activity, we used a generalized estimating equation (GEE) model, applied to all patients either with or without active disease. According to this analysis, CAR and NLR baseline levels were predictive of higher risk of disease activity at 6 months follow up (*p* < 0.001).

**Conclusions:**

CAR and NLR could indicate persistent disease activity in patients with JIA. Their predictive value could be increased by their combined use and by the observation of their trend during follow up, since increasing CAR values over time could predict a disease flare in the brief time.

**Supplementary Information:**

The online version contains supplementary material available at 10.1186/s41927-024-00390-x.

## Introduction

Juvenile idiopathic arthritis (JIA) is the most prevalent chronic rheumatic disease in children and an important cause of short-term and long-term disability and reduced quality of life. In the last two decades, improved knowledge of JIA pathogenesis has allowed the development of targeted therapies and combination treatment strategies [1]. Thanks to these advances, about 46–57% of patients with JIA attain clinical remission within 5 years, except for children with polyarthritis [2]. Although many patients can discontinue treatment, up to 50% of early flares after drug discontinuation has been reported in the literature [3, 4]. Therefore, the identification of new biomarkers, which correlate with clinical and subclinical disease activity, is an important goal for children with JIA and an open field of scientific research. The incorporation of reliable standardized biomarkers in clinical care may allow the clinicians to better design patient-tailored treatment regimens and it may help to choose the optimal modalities and timing for treatment discontinuation [5].

Recent scientific works pointed out the role of new derivative indices as biomarkers of systemic inflammation and disease activity in patients with autoimmune diseases, including rheumatoid arthritis (RA). Studies on adult RA patients have shown a positive correlation between C Reactive Protein (CRP) to Albumin Ratio (CAR) and disease activity and risk of flare [6–8]. Moreover, He Y. et al. demonstrated a correlation between CAR and Th17 cells and cytokine expression in patients with RA [9]. Other authors reported similar results in adult patients with axial spondyloarthritis (axSpA) [10] and psoriasic arthritis (psA) [11]. Besides, several reports have suggested the potential role of neutrophil to lymphocyte ratio (NLR) and platelet to lymphocyte ratio (PLR) as inflammatory markers and complementary diagnostic tools in RA and other rheumatic diseases [12, 13]. Interestingly, Slouma et al. reported a positive correlation between PLR and other indices, like CAR, CRP, and erythrocyte sedimentation rate (ESR), and between PLR, CAR, and disease activity in adult patients with axSpA. Therefore, CAR and PLR could be used together to identify patients with high disease activity [14]. Furthermore, RA patients with increased platelet count (higher PLR) seem to present more substantial improvement in disease activity score 28 (DAS28) and acute-phase reactants after treatment with biologics, like anti-IL6 tocilizumab [15]. Little is known about the role of CAR, PLR and NLR in pediatric inflammatory diseases. Li W. et al. conducted a retrospective study on 186 children with newly diagnosed juvenile systemic lupus erythematosus (jSLE). These authors found that PLR and NLR had correlations with serological indicators, like anti-Smith (anti-Sm) antibodies, and could predict organ involvement [16]. The role of CAR, NLR and PLR as markers of disease activity has never been evaluated in JIA patients.

The first aim of this work was to investigate the role of CAR, PLR and NLR as potential markers of disease activity in patients with non-systemic JIA (nsJIA). The second endpoint was to longitudinally investigate the relationship between CAR, PLR and NLR and the risk of flare or persistent disease activity during 18 months follow up.

## Materials and methods

### Study design

**Fig. 1:**
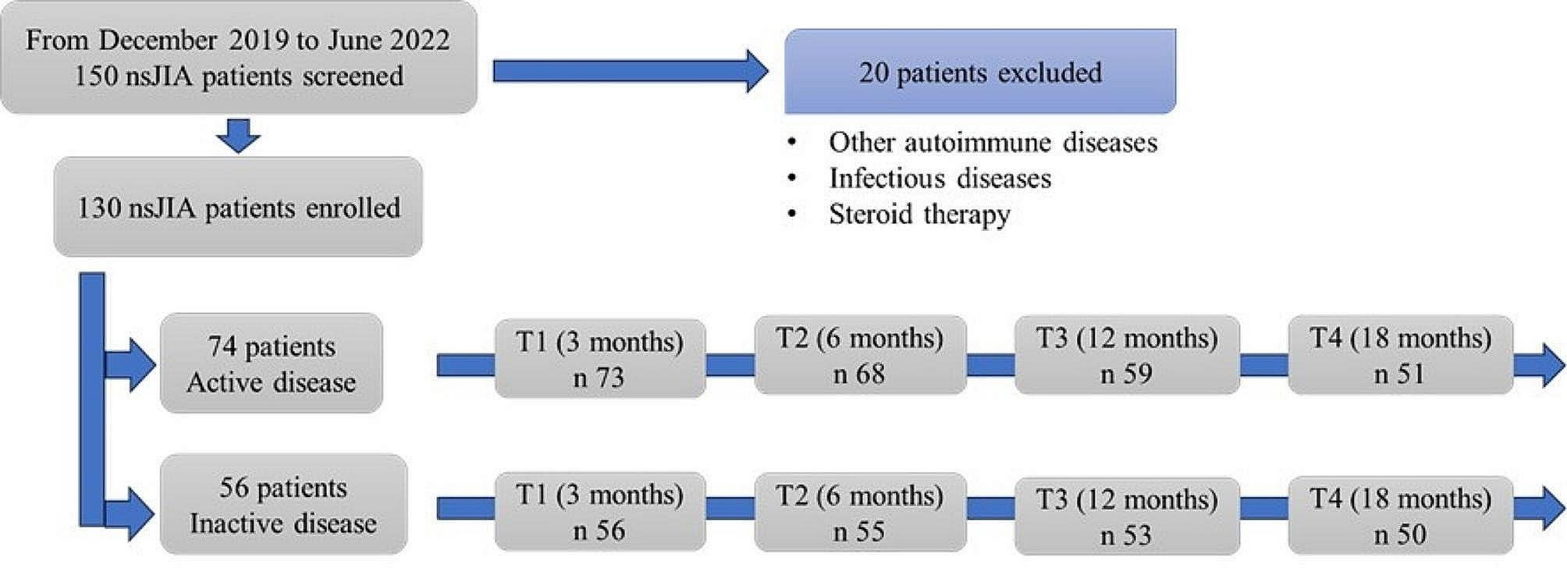
Flow chart of the study. nsJIA: non-systemic juvenile idiopathic arthritis

We performed a prospective, cross-sectional study and we involved a group of JIA patients who referred to the Rheumatology Unit of the Department of Pediatrics, University of Chieti, Italy, from December 2019 to June 2022. Patients with any subtypes of JIA, except systemic JIA (sJIA), were eligible for this study. All patients fulfilled the ILAR classification criteria for JIA [17]. The exclusion criteria were the following: sJIA, steroid therapy at the time of the enrollment time or in the previous 2 weeks, infectious disease at the time of the enrollment or in the previous 2 weeks and associated autoimmune diseases. The case study population included 103 females (79%) and 27 males (21%), with a mean age of 11 ± 5 years. Of the total 130 JIA patients, 74 had clinically active disease, while 56 had inactive disease according to Wallace criteria [18]. We also included in the study 62 healthy controls (31 males and 31 females, mean age 10 ± 5 years). The exclusion criteria for healthy controls were the following: autoimmune or autoinflammatory diseases, immunodeficiencies, hematologic conditions, malignances, steroid therapy at the time of the enrollment or in the previous 2 weeks, infectious disease at enrollment time or in the previous 2 weeks. We performed a second longitudinal study phase on the patient group, with evaluation time-points at 3 months (T1), 6 months (T2), 12 months (T3) and 18 months (T4) from baseline (T0). During the longitudinal phase in the follow up, not all patients maintained the same therapeutic regimen as at baseline. Of all 74 active patients, 73 patients reached the first time-point (T1), 68 reached T2, 59 reached T3 and 51 were evaluated at 18 months follow up (31% lost to follow up). Of all 56 inactive patients, the same 56 patients reached the first time-point (T1), 55 reached T2, 53 reached T3 and 50 were evaluated at 18 months follow up (11% lost to follow up). The flow-chart of the study is shown in Fig. 1.

The present study was approved by the local Ethics Committee. All procedures involving human biological material followed the ethical standards of Helsinki. Written informed consent was obtained from all participating subjects or their parents/guardians.

### Demographic and clinical assessment

At baseline visit (T0), the following data were recorded for each patient: age, sex, weight, body mass index (BMI), BMI SDS, age at disease onset, disease duration, JIA category, the number of involved joints at baseline and at disease onset, anti-nuclear antibodies (ANA), rheumatoid factor (RF) and HLA-B27 status and history of previous uveitis. Age, sex, BMI and BMI SDS were also reported for healthy controls at baseline. Furthermore, information about therapeutic interventions were collected at baseline for all patients: number of previous intra-articular corticosteroid injections (IACIs), JIA medications received prior and at study entry, Methotrexate (MTX) and biologic therapy duration. For each patient, 27 joints were assessed for signs of inflammation (swelling, tenderness, pain on motion and restricted motion) and disease activity was evaluated at baseline and at each time-point through the Juvenile Arthritis Disease Activity Score (JADAS-27), a validated score adopting four criteria: number of swallen joints (0–27), physician global assessment of disease activity (0–10), parent’s/patient’s assessment of child’s well-being (0–10), and ESR. ESR value was normalized to a 0–10 scale according to the following formula: (ESR (mm/h) – 20)/10. The JADAS-27 was calculated as the simple linear sum of the scores of its components, allowing a global score of 0–57, 0 corresponding to inactive disease and 57 to maximum disease activity. Inactive disease was defined according to Wallace criteria, as the presence of no joints with active arthritis, no fever, rash, serositis, splenomegaly or generalized lymphadenopathy attributable to JIA, no active uveitis, normal CRP and ESR, and a physician global assessment indicating no disease activity. Clinical remission on therapy was attributed to patients with inactive disease for a continuous period of 6 months, while clinical remission off therapy was defined in patients with inactive disease for a minimum of continuous 12 months while off all anti-arthritis and anti-uveitis medications [18].

### Laboratory assessment

Morning blood samples were collected from patients and healthy controls after 12 h of fasting. Complete blood count, acute phase reactants (ESR and CRP), serum ferritin and albumin were measured in active and inactive JIA patients and in healthy controls at T0 and at every time-point in the longitudinal phase. CRP was measured with a quantitative immune-turbidimetric assay, with normal values (n.v.) in the range of 0.00–10.00 mg/l. ESR was calculated using the Westergren technique (n.v. 0–15 mm/h in males and 0–20 mm/h in females). Ferritin was measured with a quantitative chemiluminescence immunoassay (n.v. 4.6–204 ng/ml). Albumin was measured with a colorimetric assay. At each time-point, CAR, NLR and PLR were calculated as the simple ratio between CRP and albumin, neutrophil and lymphocyte counts, and platelet and lymphocyte counts respectively.

### Statistical analysis

Continuous data was expressed as mean (standard deviation (SD)) or median (5–95% range), and categorical data was presented as percentage and count. Shapiro-Wilk test was used to evaluate whether continuous data was normally distributed. We compared characteristics of the study groups by using Anova one-way tests, Kruskall Wallis tests, and Pearson’s Chi-square tests. Bonferroni correction test was used both to correct the experiment-wise error rate when we performed multiple ‘t’ tests and as a post-hoc procedure to correct the family-wise error rate following analysis of variance (Anova). We performed Spearman’s correlation to evaluate the relationship of disease activity, measured by JADAS-27, with CAR, NLR, and PLR ratios. We used a Generalized Estimating Equation (GEE) model to investigate longitudinally the associations of CAR, PLR, NLR ratios with disease activity in JIA patients across a 18-months follow up. We used independent correlation structure for that model. Additionally, we adjusted our model for potential confounders, such as gender, BMI SDS, age at the baseline, disease duration, biologic therapy duration. Confounders were selected from literature. A p value < 0.05 was considered statistically significant. Statistical analyses were performed using SPSS version 25.0 for Windows software (IBM Corp) and STATA/IC 15.1 (StataCorp LLC 4905 Lakeway Drive College Station, Texas 77,845 − 4512, USA), and GraphPad Prism version 4.00 for Windows, GraphPad Software, San Diego California USA, “www.graphpad.com”.

## Results

The whole “case” study group included 130 JIA patients with a higher prevalence of girls (103 females, 79%). The mean age of JIA patients was 11 ± 5 years. Among all patients, 17 (23%) were enrolled at disease onset. Ninety-two patients (71%) were on therapy at enrollment, while 38 (29%) were off therapy. Of all treated patients, 30 were receiving MTX (23%), 22 etanercept (17%), 26 adalimumab (20%) as monotherapy, while the remaining patients were on MTX-biologic combinations or non-steroid anti-inflammatory drugs (NSAIDs). The “control” group included 62 healthy children, without sex prevalence (females = 31, 50%). The mean age of the control group was 10 ± 5 years. There were no relevant differences in anthropometric and demographic variables between the two groups, except for sex, with a female prevalence in JIA patients (*p* < 0.001), as expected from the literature [19]. CRP values showed a statistically significant difference between patients and controls (*p* = 0.046), as did CAR (*p* = 0.046), with higher levels in the study group (Figure S1). On the other hand, ferritin levels were found to be statistically higher in the control group (*p* = 0.003). No differences were found for NLR and PLR between the two groups. Demographic, clinical and laboratory characteristics of the study population at baseline are resumed in Table 1.

**Table 1:** Baseline characteristics of JIA study population and healthy controls

	JIA patients (*n* = 130)	Control group (*n* = 62)	*p*-value
Sex, female (n(%))	103.0 (79.0)	31.0 (50.0)	**< 0.001**
Age (yr), mean (SD)	11.0 (5.0)	10.0 (5.0)	0.567
Weight (kg), mean (SD)	39.0 (18.0)	40.5 (20.9)	0.616
Height (cm), mean (SD)	138.0 (25.0)	142.5 (28.0)	0.731
BMI (kg/m^2^), mean (SD)	19.0 (4.0)	19.7 (4.7)	0.273
BMI SDS, mean (SD)	-0.03 (1.0)	0.05 (1.3)	0.659
ANA positive(n(%))	100.0 (77.0)	-	
RF positive (n(%))	3.0 (2.0)	-	
HLA-B27 positive(n(%))	2.0 (1.5)	-	
ESR (mm/h), median (range)	8.0 (2.0–88.0)	7.0 (2.0–31.0)	0.142
CRP (mg/l), median (range)	0.8 (0.1–32.0)	0.5 (0.1–9.9)	**0.046**
Hb (g/dl), mean (SD)	13.0 (1.1)	13.2 (1.2)	0.134
PLT/ µl, median (range)	299500.0 (151000.0-524000.0)	289500.0 (176000.0-522000.0)	0.945
WBC/µl, median (range)	6735.0 (3249.0-13160.0)	7095.0 (4390.0-14420.0)	0.150
Neutrophils/µl, median (range)	3005.0 (3075.0-9370.0)	3255.0 (1430.0-7850.0)	0.116
Lymphocytes/µl, median (range)	2675.0 (760.0-6580.0)	2810.0 (1070.0-7400.0)	0.547
Ferritin (ng/mL), median (range)	22.3 (1.0-133.0)	37.1 (6.0-110.0)	**0.003**
Albumin (g/dl), mean (SD)	4.3 (0.3)	4.3 (0.2)	0.852
CAR, median (range)	0.2 (0.0-7.8)	0.1 (0.0-2.3)	**0.046**
PLR, median (range)	110.3 (52.6-319.7)	108.7 (46.1-259.8)	0.404
NLR, median (range)	1.1 (0.1–4.5)	1.1 (0.3–4.2)	0.347
Age at disease (JIA) onset, median (range)	4.6 (1.0–15.0)	-	
Disease duration (yr), mean (SD)	5.0 (4.0)	-	
**JIA categories (n(%))**		-	
• *Oligoarticular*	75.0 (58.0)		
• *Extended oligoarticular*	19.0 (15.0)		
• *Polyarticular*	27.0 (21.0)		
• *Psoriasic*	8.0 (6.0)		
• *Enthesitis-Arthritis*	1.0 (1.0)		
**Previous therapy (n(%))**		-	
• *No*	18.0 (14.0)		
• *Methotrexate*	64.0 (49.0)		
• *Etanercept*	4.0 (3.0)		
• *Adalimumab*	1.0 (1.0)		
• *MTX + Etanercept*	16.0 (12.0)		
• *MTX + Adalimumab*	6.0 (5.0)		
• *MTX + Etanercept + Adalimumab*	1.0 (1.0)		
• *NSAIDs*	17.0 (13.0)		
• *Tocilizumab*	3.0 (2.0)		
• *Intra-articular corticosteroids*	92.0 (71.0)		
Number of previous intra-articular infiltrations, median (range)	1.0 (0.0–15.0)	-	
Lag time to MTX initiation (yr), median (range)	0.3 (0.0-9.4)	-	
MTX treatment duration (yr), median (range)	1.0 (0.0-12.4)	-	
Lag time to biologic therapy initiation (yr), median (range)	0.3 (0.0-11.3)	-	
Biologic therapy duration (yr), median (range)	0.1 (0.0–13.0)	-	
On therapy (n(%))	92.0 (71.0)	-	
**Ongoing therapy (n(%))**		-	
• *None therapy or drug*	38.0 (29.0)		
• *Methotrexate*	30.0 (23.0)		
• *Etanercept*	22.0 (17.0)		
• *Adalimumab*	26.0 (20.0)		
• *MTX + Etanercept*	3.0 (2.0)		
• *MTX + Adalimumab*	3.0 (2.0)		
• *Other*	2.0 (2.0)		
• *NSAIDs*	6.0 (5.0)		
Number of involved joints at JIA onset, median (range)	2.0 (0.0–15.0)	-	
Number of involved joints at the baseline, median (range)	3.0 (0.0–15.0)	-	
History of uveitis, n(%)	28.0 (22.0)	-	

Data were expressed as mean (SD) or median and range (5–95%), or absolute numbers and percentages (%). N: number; JIA: juvenile idiopathic arthritis; yr: years; BMI: body mass index; BMI SDS: BMI standard deviation score; ANA: antinuclear antibody; RF: rheumatoid factor; HLA- B27: Human Leukocyte antigen-B27; ESR: erythrocyte sedimentation rate; CRP: C reactive protein; Hb: hemoglobin; PLT: platelets; WBC: white blood count; CAR: C reactive protein to albumin ratio; PLR: platelet to lymphocyte ratio; NLR: neutrophil to lymphocyte ratio; MTX: methotrexate; NSAIDs: non steroid anti-inflammatory drugs

Patients were divided into two subgroups: patients with active disease (*n* = 74) and patients with inactive disease (*n* = 56). Demographic and clinical data of active patients, inactive patients and controls are resumed in Table 2. There were no relevant differences in anthropometric and demographic variables between the three groups, except for sex, with a female prevalence in active and inactive patients (*p* < 0.001). We found a statistically significant difference in disease duration (*p* = 0.012), previous therapy (*p* = 0.005), history of articular infiltrations (*p* = 0.001) and biologic therapy duration (*p* = 0.019) between active and inactive JIA patients.

**Table 2:** Demographic and clinical characteristics of active patients, inactive patients, and healthy controls at baseline

	Active disease (*n* = 74)	Non active disease (*n* = 56)	Control group (*n* = 62)	*p*-value
Sex, female (n(%))	57.0 (77.0)	46.0 (82.0)	31.0 (50.0)	**< 0.001**
Age (yr), mean (SD)	10.3 (5.7)	10.7 (3.9)	10.0 (5.0)	0.764
Weight (kg), mean (SD)	37.8 (19.4)	40.6 (16.5)	40.5 (20.9)	0.634
Height (cm), mean (SD)	147.7 (28.1)	144.2 (21.4)	142.5 (28.0)	0.448
BMI (kg/m^2^), mean (SD)	18.7 (3.7)	19.3 (3.5)	19.7 (4.7)	0.380
BMI SDS, mean (SD)	-0.1 (1.1)	0.01 (1.0)	0.05 (1.3)	0.650
Age of JIA onset (yr), median (range)	4.6 (1.0–15.0)	4.4 (1.0-14.5)		0.388
Disease duration (yr), mean (SD)	4.3 (4.7)	5.4 (3.8)		**0.012**
**JIA categories (n(%))**				0.794
• *Oligoarticular*	44.0 (59.5)	31.0 (55.4)		
• *Extended oligoarticular*	9.0(12.2)	10.0(17.9)		
• *Polyarticular*	15.0(20.3)	(21.4) 12.0		
• *Psoriasic*	5.0 (6.8)	3.0 (5.4)		
• *Enthesitis-Arthritis*	1.0 (1.4)	0.0 (0.0)		
**Previous therapy (n, (%))**				**0.005**
• *None therapy or drug*	17.0	1.0 (1.8)		
• *Methotrexate*	35.0	29.0(51.8)		
• *Etanercept*	4.0(5.4)	0.0 (0.0)		
• *Adalimumab*	1.0 (1.4) (47.3)	0.0 (0.0)		
• *MTX + Etanercept*	7.0 (9.5)	9.0 (16.1)		
• *MTX + Adalimumab*	3.0 (4.1)	3.0 (5.4)		
• *MTX + Etanercept + Adalimumab*	1.0 (1.4)	0.0 (0.0)		
• *NSAIDs*	5.0 (6.8)	12.0 (21.4)		
• *Tocilizumab*	1.0 (1.4)	2.0 (3.6)		
• *Intra-articular corticosteroids*	44.0(59.5)	48.0 (85.7)		
Number of previous intra-articular infiltrations, median (range)	1.0 (0.0–15.0)	1.0 (0.0–5.0)		0.140
Lag time to MTX initiation (yr), median (range)	0.2 (0.0-6.2)	0.3 (0.0-9.4)		0.431
MTX treatment duration (yr), median (range)	0.7 (0.0-12.4)	1.3 (0.0-6.9)		0.068
Lag time to biologic therapy initiation (yr), median (range)	0.1 (0.0-8.6)	0.3 (0.0-11.3)		0.864
Biologic therapy duration (yr), median (range)	0.1 (0.0–13.0)	0.7 (0.0–9.0)		**0.019**
On therapy (n(%))	53.0 (71.6)	39.0 (69.6)		0.523
**Ongoing therapy (n(%))**				0.071
• *No*	21.0 28.4)	17 (30.3)		
• *Methotrexate*	17.0(23.0)	13.0 (23.2)		
• *Etanercept*	10.0(13.5)	12.0 (21.4)		
• *Adalimumab*	12.0(16.2)	14.0 (25.0)		
• *MTX + Etanercept*	3.0 (4.1)	0.0 (0.0)		
• *MTX + Adalimumab*	3.0 (4.1)	0.0 (0.0)		
• *Other*	2.0 (2.7)	0.0 (0.0)		
• *NSAIDs*	6.0(8.1)	0.0 (0.0)		
Number of involved joints at JIA onset, median (range)	2.0 (1.0–15.0)	2.0 (0.0–14.0)		0.143
History of uveitis (n(%))	16.0 (21.6)	12.0 (21.4)		0.678
ANA positive^+^ (n(%))	56.0 (75.7)	44.0 (78.6)		0.432
RF positive^+^ (n(%))	1.0 (1.4)	2.0 (3.6)		**< 0.001**
HLA-B27 positive^+^ (n(%))	2.0 (2.7)	0.0 (0.0)		**< 0.001**
JADAS 27, median (range)	5.0 (0.5–20.0)	0.0 (0.0-5.4)		**< 0.001**

Data were expressed as mean (SD) or median and range (5–95%), or absolute numbers and percentages (%). N: number; JIA: juvenile idiopathic arthritis; yr: years; BMI: body mass index; BMI SDS: BMI standard deviation score; ANA: antinuclear antibody; RF: rheumatoid factor; HLA- B27: Human Leukocyte antigen-B27; JADAS-27: juvenile arthritis disease activity score-27; MTX: methotrexate; NSAIDs: non steroid anti-inflammatory drugs

Laboratory assessments of active patients, inactive patients and controls at baseline are summarized in Table 3. No significant differences were found in ESR, CRP, whole blood count (WBC), hemoglobin, neutrophils, and lymphocytes levels between active patients, inactive patients, and controls. Platelet count was increased in active patients as compared to inactive ones (*p* = 0.028). Furthermore, albumin values showed a statistically significant difference between active and inactive patients (*p* < 0.001), active patients and controls (*p* = 0.026), inactive patients and controls (*p* = 0.001). However, no differences were found in CAR, NLR and PLR values between the three groups (Figure S2). Besides, there was no positive correlation between CAR (R2 = 0.004; *p* = 0.475), NLR (R2 = 0.0014; *p* = 0.674), PLR (R2 = 0.003; *p* = 0.535) and JADAS-27 in JIA patients (Figure S3). Therefore, these indices did not correlate with disease activity in our cohort of nsJIA patients.

**Table 3:** Laboratory evaluations of active patients, inactive patients and healthy controls at baseline

	Active disease (*n* = 74)	Non active disease (*n* = 56)	Control group (*n* = 62)	*p*-value
ESR (mm/h), median (range)	8.0 (2.0–88.0)	7.5 (2.0–64.0)	7.0 (2.0–31.0)	0.124
CRP (mg/l), median (range)	0.6 (0.1–32.0)	0.9 (0.1–25.6)	0.5 (0.1–9.9)	0.083
Hb (g/dl), mean (SD)	12.8 (1.3)	13.2 (0.8)	13.2 (1.2)	0.088
PLT/ µl, median (range)	325000.0 (178000.0-524000.0)	287500.0 (151000.0-494000.0)	289500.0 (176000.0-522000.0)	**0.034**
WBC/µl, median (range)	6735.0 (3249.0-13160.0)	6735.0 (3530.0-10110.0)	7095.0 (4390.0-14420.0)	0.169
Neutrophils/µl, median (range)	3140.0 (375.0-9370.0)	2795.0 (1070.0-6930.0)	3255.0 (1430.0-7850.0)	0.199
Lymphocytes/µl, median (range)	2825.0 (1029.0-6580.0)	2550.0 (760.0-5280.0)	2810.0 (1070.0-7400.0)	0.500
Ferritin (ng/mL), median (range)	21.8 (1.0-133.0)	24.6 (2.0–83.0)	37.1 (6.0-110.0)	**0.023**
Albumin (g/dl), mean (SD)	4.2 (0.2)	4.4 (0.2)	4.3 (0.2)	**< 0.001**
CAR, median (range)	0.1 (0.0-7.7)	0.2 (0.0-5.6)	0.1 (0.0-2.3)	0.103
PLR, median (range)	110.8 (55.8-319.7)	110.3 (52.6-269.7)	108.7 (46.1-259.8)	0.921
NLR, median (range)	1.2 (0.1–4.5)	1.1 (0.3–4.3)	1.1 (0.3–4.2)	0.797

Data were expressed as mean (SD) or median and range (5–95%), or absolute numbers and percentages (%). N: number; ESR: erythrocyte sedimentation rate; CRP: C reactive protein; Hb: hemoglobin; PLT: platelets; WBC: white blood count; CAR: C reactive protein to albumin ratio; PLR: platelet to lymphocyte ratio; NLR: neutrophil to lymphocyte ratio

**Fig. 2:**
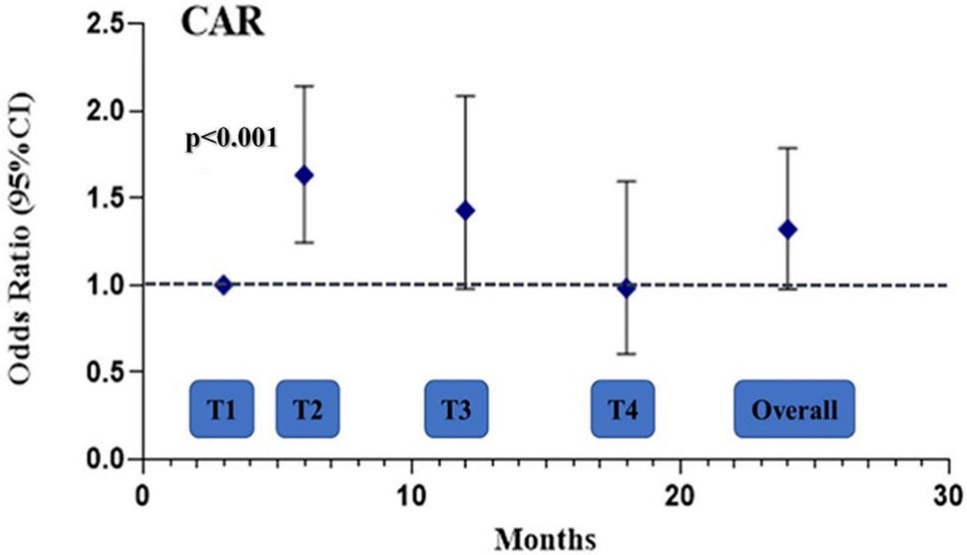
Odds ratio with 95% confidence intervals of CAR for persistent disease activity at each timepoint and overall from GEE adjusted model. CI: confidence interval; CAR: C reactive protein to albumin ratio; GEE: generalized estimated equation

**Fig. 3:**
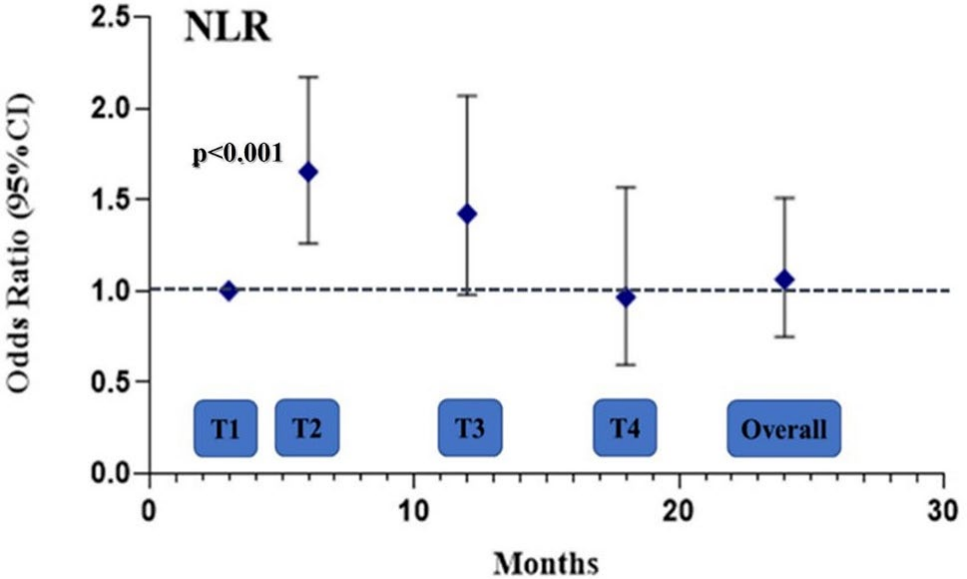
Odds ratio with 95% confidence intervals of NLR for persistent disease activity at each timepoint and overall from GEE adjusted model. CI: confidence interval; NLR: neutrophil to lymphocyte ratio; GEE: generalized estimated equation

During the longitudinal phase of the study, all JIA patients were evaluated at 4 time-points: 3 (T1), 6 (T2), 12 (T3) and 18 months (T4). The measured endpoint was disease activity at each time-point (JADAS-27 > 1). Twenty-nine inactive patients (51.7%) presented a disease flare while 35 active patients (47.3%) maintained a persistent disease activity during follow up. To better investigate the role of CAR, NLR and PLR as markers of disease activity, we used a GEE model for Repeated Measures Analysis, applied to the whole group of JIA patients (both active and inactive at baseline). Interestingly, we found that patients with higher CAR values at baseline showed a higher risk of disease activity at 6 months of follow up (odds ratio OR (CI95%), 1.6 (1.2, 2.1)), although there was no association of CAR values at baseline with the overall odds ratio (Fig. 2). Similarly, we found that patients with higher NLR values at baseline showed a higher risk of disease activity at 6 months of follow up (odds ratio OR (CI95%), 1.7 (1.3, 2.2)), although there was no association of NLR at baseline with the overall odds ratio (Fig. 3). Differently, no associations of PLR values at baseline with the odds ratio of disease activity at every time point were found.

## Discussion

To the best of our knowledge, CAR, NLR and PLR predictive role in JIA has never been investigated in the literature. In our study, we analyzed the demographic, clinical and laboratory characteristics of a group of children affected by nsJIA, compared to age-matched healthy controls. We found that patients have higher CAR levels as compared to controls, and that patients with higher CAR and NLR values at baseline show a higher risk of disease activity at 6 months of follow up.

CRP is synthesized by the liver and reflects systemic inflammation, so that it is widely used as inflammatory indicator in clinical practice. Albumin is an indicator of nutritional status, which is reduced in case of severe malnutrition, chronic inflammation, and autoimmune diseases. Therefore, integrating the effects of CRP and albumin, CAR could be a reliable marker of inflammation. Recently, it has emerged as a novel biomarker for evaluating disease activity in some rheumatic diseases [6–11]. Furthermore, growing evidence are pointing out the potential prognostic value of blood cell count ratios, like NLR and PLR, as non-specific inflammatory markers [12, 13].

The first endpoint of our study was to evaluate the relationship between CAR, NLR, PLR and disease activity in JIA. The second was to investigate the relationship between CAR, PLR and NLR and the risk of persistent disease activity during follow up. CRP and CAR levels were found to be significantly higher in JIA patients as compared to controls, as reported in previous studies on adult patients with RA, axSpA and psA [8, 10, 11]. Otherwise, ESR, PLR and NLR were not different between the two groups. To better understand the relationship of CAR, NLR, PLR and disease activity, we compared the clinical and laboratory characteristics of active and inactive patients: the classic inflammatory markers (CRP, ESR) and the new derivative indices (CAR, NLR and PLR) were not different between the three groups. Furthermore, we found no correlation between CAR, NLR, PLR and disease activity, represented by JADAS-27 score. Our results are in contrast with previous studies, showing increased CAR levels in active RA patients, and a positive correlation between CAR and disease activity scores (DAS-28) [7]. RA is a very different disease from JIA. In fact, in adult patients, CRP and ESR values are important for diagnosis and disease activity assessment. Particularly, CRP seems to be directly involved in RA pathogenesis, since it seems to activate fibroblast-like synoviocytes (FLSs) via CD32/64-p38 and NF-κB signaling pathways [20]. Therefore, CRP levels are strictly related to synovial inflammation and disease activity. In RA patients, CRP is a reliable marker of systemic inflammation, and its levels appear to be related with the risk of associated comorbidities, like cardiovascular diseases, diabetes, metabolic syndrome, pulmonary diseases, and depression [21]. It derives that CAR, which is influenced by CRP, could be a reliable laboratory indicator of inflammation and disease activity in RA. Differently, JIA is not a single disease, as it encompasses a heterogeneous spectrum of clinical manifestations, with different pathogenic mechanisms, prognosis, and outcome. Besides, acute-phase reactants are often normal or moderately increased in JIA, and they do not correlate with disease activity [22].

Similarly, PLR and NLR seem to have a different predictive value in the pediatric age, as compared to the adult counterpart. In fact, NLR and PLR have been found to be increased in RA patients [12], differently from our study. Zhong Z. et al. also described increased CAR, NLR and PLR levels in active axSpA patients and found a positive correlation between CAR and Bath Ankylosing Spondylitis Activity Index (BASDAI), which are in contrast with our results. The authors had also proposed an optimal cut-off value of CAR for active axSpA patients (0.3644). However, as in our study, they did not find a correlation between NLR, PLR and Bath Ankylosing Spondylitis Disease Activity Index (BASDAI) [10]. Differently, Slouma et al. found a positive correlation between PLR and Ankylosing Spondylitis Disease Activity Score-CRP (ASDAS-CRP) [14]. Platelets are rich in proinflammatory agents and release highly active microparticles, that can interact with neutrophils via the expression of platelet-type lipoxygenase and activation of the eicosanoid pathway [23]. It has been shown that synovial neutrophils of RA patients internalize platelet microparticles, which intensify synovial inflammation. Moreover, anti-tumor necrosis factor (TNF) α treatment seems to reduce the ability of platelets to bind to and activate leukocytes in RA, which may decrease the risk of thrombotic events [24, 25]. Interestingly, observational studies have proved that platelet counts gradually increase with radiological disease progression in RA, while leukocyte count variations are unremarkable [26]. Moreover, RA patients with increased platelet count show more substantial improvements in DAS28 and acute-phase reactants in response to tocilizumab, than do RA patients with normal platelet counts [15]. Therefore, platelet count may indicate autoimmune disease activity, response to anti-inflammatory therapies, and the presence of various comorbidities. However, platelet count presents important fluctuations, due to their migration to and excessive consumption at inflammatory sites, and their destruction via binding to anti-platelet antibodies. The stress-induced hypercortisolemia with subsequent platelet release and transient lymphopenia influence the degree of PLR elevation. PLR value as an inflammatory marker seems to increase when its fluctuations are interpreted along with other complementary hematologic indices, particularly NLR. In fact, PLR and NLR have shown high predictive value in rheumatic diseases with predominantly neutrophilic inflammation, like Behçet disease and familial mediterranean fever [27]. The role of these indices in pediatric autoimmune diseases has never been investigated. JIA is a lymphocyte-mediated autoimmune disease, with a central role of T cells and autoantibodies and a heterogeneous circulating lymphocytes profile, according to disease category [28]. Furthermore, growing evidence suggests an important role for neutrophils in JIA pathogenesis. In fact, some authors reported that synovial fluid neutrophils display an activated, hypersegmented phenotype in JIA patients [29]. However, platelet, lymphocyte and neutrophil counts are extremely variable in the pediatric population, since they are race, age, and sex dependent. Moreover, single measures of laboratory parameters do not reflect their dynamics and are influenced by many confounding factors (nutritional state, anemia, intercurrent infectious diseases, associated autoimmune diseases and ongoing treatments). Therefore, the reliability of these indices should be interpreted based on the specific inflammatory condition, the ongoing therapy, and the associated comorbidities. Their value could also be increased by their combined use and by the evaluation of their trend over time.

This study has some limitations. First, it was a single-center study, with a relatively small sample size. Second, patients enrolled in the study represented a heterogeneous population in terms of age, gender, JIA subtype, disease duration and previous/ongoing therapy. Third, we performed a prospective cross-sectional study with 18 months follow up. Unfortunately, the entire follow up was not completed by all patients, with an overall loss to follow up of 22.3%. Our study has also some strengths. To the best of our knowledge, this is the first study evaluating the relationship between CAR, NLR and PLR and disease activity in JIA. Previous studies have been only conducted on adult RA. Moreover, this is the first study evaluating the trend of these indices during a long follow up and their relationship with the risk of persistent persistent disease activity.

## Conclusions

Literature data suggest that CAR, NLR and PLR could help the clinician in the definition of disease activity and in the prevision of therapeutic response or flare risk in patients with rheumatic diseases. However, they are influenced by various confounding factors. In our work, CAR was significantly increased in JIA patients as compared to healthy subjects, without a clear correlation with disease activity scores. Moreover, according to our predictive model, basal CAR and NLR could effectively predict persistent disease activity at 6 months follow up, thus influencing clinician’s decisions about treatment discontinuation or tapering. However, significant cut-off values have not been identified and the role of these indices in the long term follow up has still to be established. Further prospective studies with a larger and more homogeneous sample and with a complete follow up should be carry out to better clarify the role of CAR, PLR and NLR in the evaluation of disease activity in JIA. Finally, their evaluation in patients with sJIA could be of great interest, given its peculiar inflammatory pathogenesis.

### Electronic supplementary material

Below is the link to the electronic supplementary material.

Supplementary Material 1

Supplementary Material 2

Supplementary Material 3

## Data Availability

The datasets generated and/or analyzed during the current study are available from the corresponding author on reasonable request.

## Abbreviations

ANA: Anti-nuclear antibodies
Anti-Sm: Anti-Smith antibodies
ASDAS-CRP: Ankylosing Spondylitis Disease Activity Score-CRP
axSpA: Axial spondyloarthritis
BASDAI: Bath Ankylosing Spondylitis Disease Activity Index
BMI: body mass index
CAR: C reactive protein to albumin ratio
CI: Confidence interval
CRP: C reactive protein
DAS28: Disease activity score 28
ESR: Erythrocyte sedimentation rate
FLSs: Activate fibroblast-like synoviocytes
GEE: Generalized Estimating Equation
IACIs: Intra-articular corticosteroid injections (IACIs)
JADAS-27: Juvenile Arthritis Disease Activity Score
JIA: Juvenile idiopathic arthritis
jSLE: Juvenile systemic lupus erythematosus
MTX: Methotrexate
NLR: Neutrophil to lymphocyte ratio
ns-JIA: Non-systemic juvenile idiopathic arthritis
OR: Odds ratio
PLR: Platelet to lymphocyte ratio
psA: Psoriasic arthritis
RA: Rheumatoid arthritis
RF: Rheumatoid factor
SD: Standard deviation
sJIA: Systemic juvenile idiopathic arthritis
TNF: Tumor necrosis factor
WBC: Whole blood count
