# Supplementary figures and images for: Associations of C reactive protein to albumin ratio, neutrophil to lymphocyte ratio, platelet to lymphocyte ratio with disease activity in patients with juvenile idiopathic arthritis
Source: BMC Rheumatol. 2024 Jun 17;8:26. doi: 10.1186/s41927-024-00390-x (PMC11181586; doi:10.1186/s41927-024-00390-x)

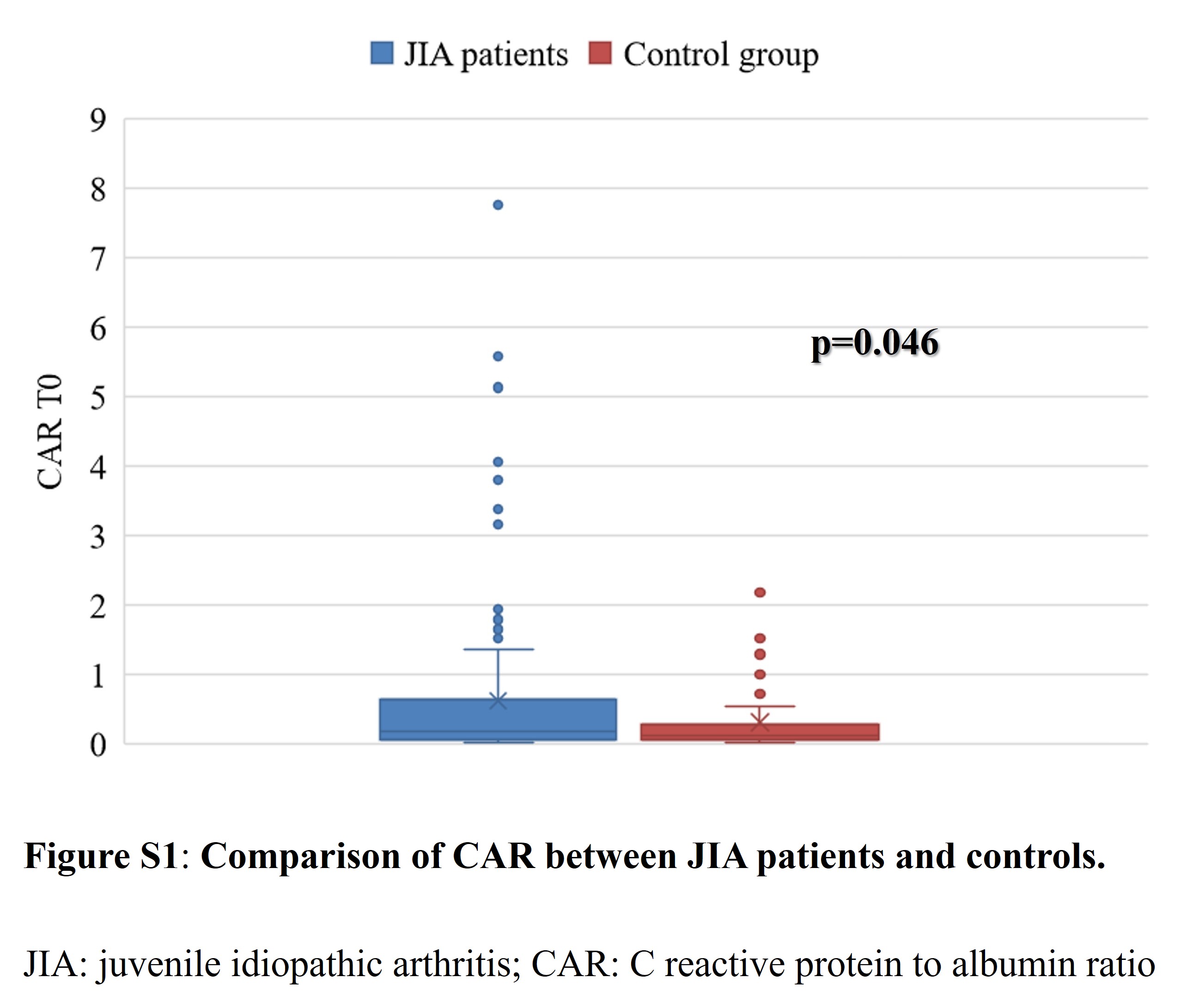

Supplement: Supplementary file 1 — Supplementary Material 1 [file 41927_2024_390_MOESM1_ESM.jpg]

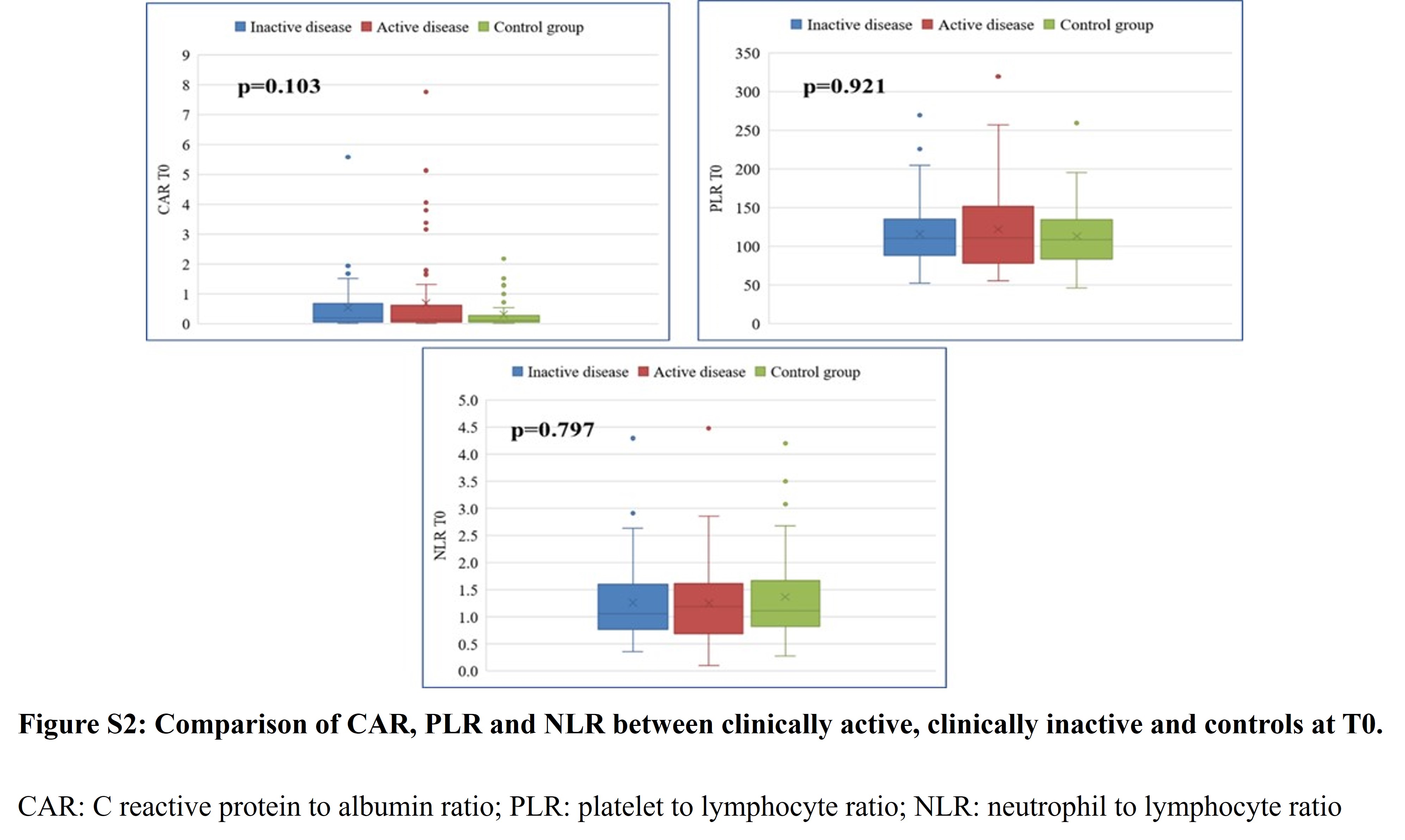

Supplement: Supplementary file 2 — Supplementary Material 2 [file 41927_2024_390_MOESM2_ESM.jpg]

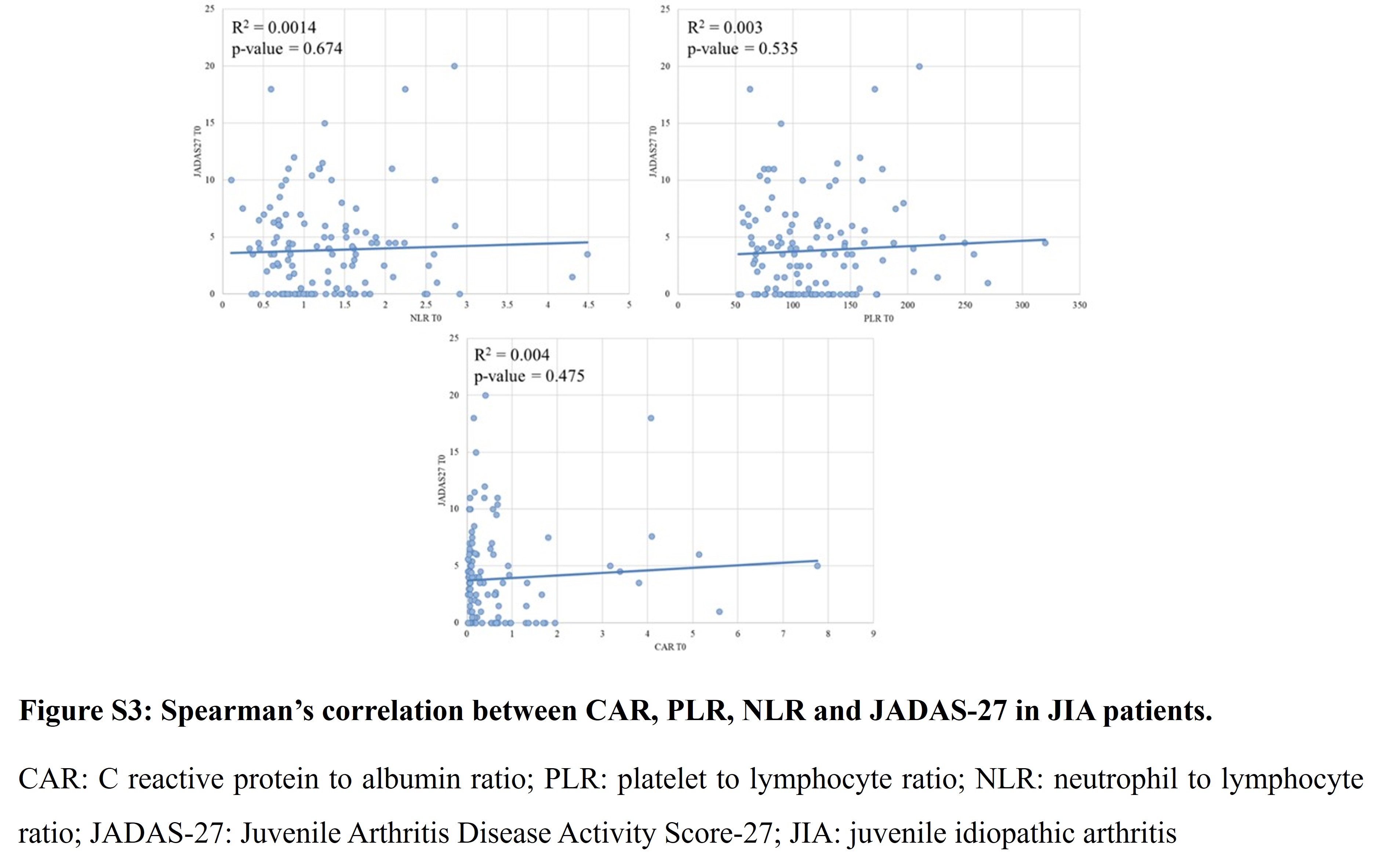

Supplement: Supplementary file 3 — Supplementary Material 3 [file 41927_2024_390_MOESM3_ESM.jpg]
